# Deconstructing cerebellar development cell by cell

**DOI:** 10.1371/journal.pgen.1008630

**Published:** 2020-04-16

**Authors:** Max J. van Essen, Samuel Nayler, Esther B. E. Becker, John Jacob

**Affiliations:** 1 Department of Physiology, Anatomy and Genetics, University of Oxford, Oxford, United Kingdom; 2 Nuffield Department of Clinical Neurosciences, University of Oxford, Oxford, United Kingdom; University College London, UNITED KINGDOM

## Abstract

The cerebellum is a pivotal centre for the integration and processing of motor and sensory information. Its extended development into the postnatal period makes this structure vulnerable to a variety of pathologies, including neoplasia. These properties have prompted intensive investigations that reveal not only developmental mechanisms in common with other regions of the neuraxis but also unique strategies to generate neuronal diversity. How the phenotypically distinct cell types of the cerebellum emerge rests on understanding how gene expression differences arise in a spatially and temporally coordinated manner from initially homogeneous cell populations. Increasingly sophisticated fate mapping approaches, culminating in genetic-induced fate mapping, have furthered the understanding of lineage relationships between early- versus later-born cells. Tracing the developmental histories of cells in this way coupled with analysis of gene expression patterns has provided insight into the developmental genetic programmes that instruct cellular heterogeneity. A limitation to date has been the bulk analysis of cells, which blurs lineage relationships and obscures gene expression differences between cells that underpin the cellular taxonomy of the cerebellum. This review emphasises recent discoveries, focusing mainly on single-cell sequencing in mouse and parallel human studies that elucidate neural progenitor developmental trajectories with unprecedented resolution. Complementary functional studies of neural repair after cerebellar injury are challenging assumptions about the stability of postnatal cellular identities. The result is a wealth of new information about the developmental mechanisms that generate cerebellar neural diversity, with implications for human evolution.

## Introduction

**Fig 1:**
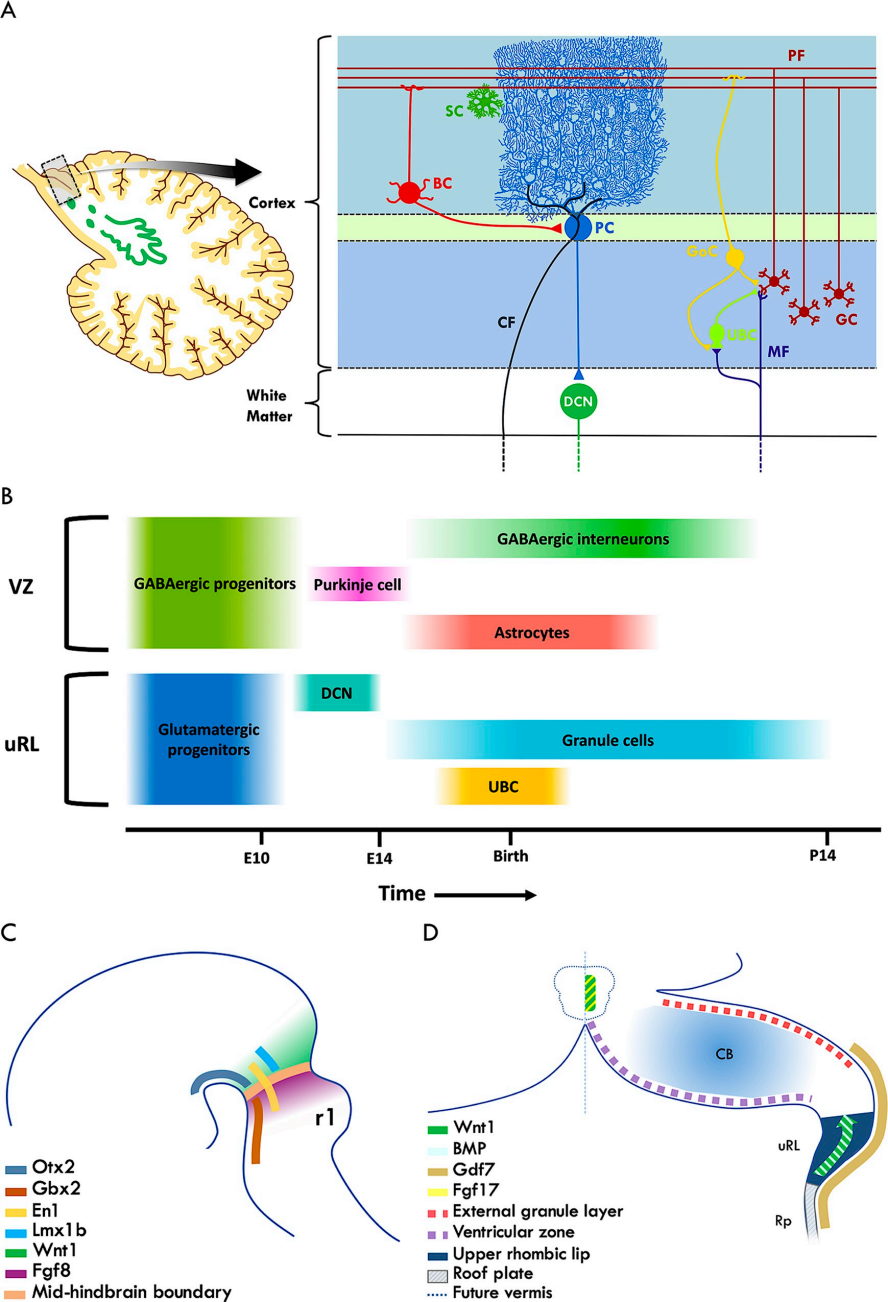
Specification of the CB and the major constituent cell types in mouse. (A) Organisation of cell types in the mature CB. Afferent input is transmitted via MFs and CFs. BC, GoC, SC, and UBC are interneuron subtypes. (B) Progenitors in two germinal zones, the VZ and uRL, produce distinct neuronal and glial cellular subtypes sequentially. (C) The future CB develops immediately posterior to the mid-hindbrain boundary. Patterning genes and secreted molecules involved in specifying this territory are indicated. (D) The Rp and cerebellar midline have important signalling functions that establish distinct regions of the CB, including the uRL and future vermis. BC, basket cell; BMP, bone morphogenetic protein; CB, cerebellum; CF, climbing fibre; DCN, deep cerebellar nuclear neuron; E, embryonic day; En1, engrailed homeobox 1; Fgf8, fibroblast growth factor 8; Fgf17, fibroblast growth factor 17; Gbx2, gastrulation brain homeobox 2; Gdf7, growth differentiation factor 7; GC, granule cell; GoC, Golgi cell; Lmx1b, LIM homeobox transcription factor 1 beta; MF, mossy fibre; Otx2, orthodenticle homeobox 2; P, postnatal day; PC, Purkinje cell; PF, parallel fibre; r1, rhombomere 1; Rp, roof plate; SC, stellate cell; UBC, unipolar brush cell; uRL, upper rhombic lip; VZ, ventricular zone; Wnt1, wingless-type MMTV integration site family, member 1.

The cerebellum is best known for its role in integrating sensory information from the periphery to guide movement and balance. Increasingly, roles in motor learning, multimodal sensory integration, cognition, emotion, and social behaviour are also recognised that are all subserved by a restricted set of neurons with stereotyped connectivity. Reflecting its participation in diverse neurocognitive tasks, abnormal cerebellar development is associated with intellectual disability, autism spectrum disorder, and attention-deficit/hyperactivity disorder [1, 2]. The mature cerebellum has three superficial cell layers, consisting of outer molecular, intermediate Purkinje cell, and inner granular layers that are separated from the deep cerebellar nuclei by interposed white matter (**Fig 1A**). Human cerebellar development extends from 30 days postconception to the second postnatal year [3, 4], whereas the human brainstem cranial nerve nuclei [5] and the latest developing neocortical region, the frontal cortex [6], are established by the first and third trimesters, respectively. Moreover, in the mouse, the cerebellum develops over 30–35 days [7]. Its protracted development makes the human cerebellum vulnerable to environmental perturbations resulting in structural abnormalities and tumours. The major cell types of the cerebellum consist of glutamatergic, GABAergic, and glial cells. Glutamatergic, excitatory cell types consist of granule, unipolar brush cell, and deep cerebellar nuclear neurons, whereas Purkinje cells, interneurons, and a contingent of deep cerebellar nuclear neurons are GABAergic, inhibitory cells. Each cell type displays complex migratory patterns to occupy defined positions in the mature cerebellum (**Fig 1A**) that are linked to its birth order from the germinal zones of the cerebellar anlage (**Fig 1B**). The current understanding of cerebellar development has largely been derived from gene expression, lineage tracing, and genetic perturbation studies in the mouse, whose cell types, lamination, circuitry, and basic foliation patterns closely resemble those in humans [7–9].

### Multiple signalling centres coordinate cerebellar patterning, growth, and midline fusion

Analysis of mouse and chick embryos reveals the cerebellum arises from the anterior hindbrain [10, 11] following the induction by the isthmic organiser of fate-determining gene expression domains that prefigure this structure [9]. Organisers are groups of cells in the embryo that share the property of being able to induce a coherent set of structures in surrounding responsive tissue [12]. Two critical determinants of regional identity, orthodenticle homeobox 2 (*Otx2*) and gastrulation brain homeobox 2 (*Gbx2*), expressed in the presumptive midbrain and hindbrain, respectively, act coordinately with fibroblast growth factor 8 (*Fgf8*) to prevent mixing of cells across the mid-hindbrain boundary [13]. Expressed immediately anterior to *Fgf8*, wingless-type MMTV integration site family, member 1 (*Wnt1*) is essential for midbrain and cerebellum development through its activation of *Fgf8* (**Fig 1C**) [8]. Notwithstanding the role of the isthmus as the most well-known organiser of the mid/hindbrain region, the roof plate of rhombomere 1 largely gives rise to the choroid plexus [14] and produces bone morphogenetic protein (BMP) and WNT signals that pattern the dorsal neural tube [15], including the rhombic lip in mouse [16].

Normal cerebellar growth and morphogenesis depends on the integrity of the primary cilium that functions as a cellular ‘antenna’. Although most cells possess primary cilia, other cell types possess specialised motile [17], or nonmotile [18], cilia. The primary cilium acts as a signalling hub, best known for its role in transducing signalling by the diffusible morphogen sonic hedgehog (SHH) [19, 20]. In the mouse, cells at the midline of the cerebellar anlage release signals that are required for the fusion of the cerebellar hemispheres and for the growth of the vermis that occupies the midline of the mature cerebellum. In particular, WNT signalling activity by nascent cerebellar midline cells is reduced by mutations of ciliary proteins, and the resulting midline fusion defect is rescued by WNT agonist drugs [21]. Whether the effect of WNT at this later stage of cerebellar development is also mediated by FGF8 is unclear.

Greater insight into the signalling events important for cerebellar midline fusion has come from refinements in single-cell RNA-sequencing (scRNA-seq) assays. Briefly, plate- and droplet-based methods differ in terms of throughput and read depth per cell, but both employ cell-specific DNA barcodes to assign transcriptomic reads to the corresponding cell [22]. Cells are then classified into groups based on their transcriptional similarity to determine the degree of heterogeneity, which allows the identification of potentially rare cell types. These studies reveal that patterning and morphogenesis of the cerebellar anlage involves a hierarchy of signalling centres whose origins can be traced to the isthmic organiser [23]. In this respect, there are broad similarities to the early patterning of the ventral neural tube, where the notochord induces a secondary signalling centre, i.e., the floor plate, which is itself a source of positional cues [24].

In the mouse, a specialised group of roof plate cells induced by the isthmic organiser termed the isthmic node come to occupy the cerebellar midline, from which they have been proposed to control the growth and patterning of the developing vermis [23, 25, 26]. scRNA-seq revealed that these cells have the genetic signature of an organising centre; they are enriched for *Wnt* pathway genes, coexpress *Fgf17*, and signal to surrounding cells of the prospective vermis to induce their proliferation (**Fig 1D**) [23]. These findings are consistent with earlier mouse genetic knockout studies, which demonstrated a requirement for *Fgf17* and *Fgf8* for the growth of the vermis, distinct from midbrain–hindbrain boundary specification [27]. Furthermore, a broader network additionally involving *Gbx2*, *Otx2*, and the chromatin modifier chromodomain helicase DNA binding protein 7 (CHD7) has been found to link midbrain–hindbrain boundary specification with downstream FGF signalling by cerebellar midline cells in mouse [28].

In keeping with the aforementioned findings, in humans, a 2.3-Mb deletion of chromosome 8p21.2–21.3 proximal to *FGF17* leads to a marked reduction in *FGF17* expression and is associated with vermis hypoplasia (Dandy–Walker malformation) [29]. In the X-linked Opitz syndrome, characterised by cerebellar midline defects, including vermis hypoplasia, the mutated gene, midline 1 (*MID1*), which encodes a ubiquitin ligase, lies genetically upstream of *FGF17* [30]. Therefore, distinct genetic programmes confined to specific cell types and locations regulate cerebellar vermis and hemisphere development. Although mouse models of vermis hypoplasia are informative, human vermis development has additional unique features that are not adequately reflected by these models. In contrast to the mouse, the rhombic lip in humans persists throughout gestation, eventually contributing granule progenitors to the posterior vermis [31]. Sporadic vermis hypoplasia in humans is associated with intellectual and motor deficits and is now known to be strongly linked to a failure of late expansion of the rhombic lip [31, 32].

### Development of cerebellar neuronal subtypes

In the mouse, the earliest born cerebellar neurons are generated from two germinal zones: the ventricular zone and upper rhombic lip, which produce GABAergic and glutamatergic neurons, respectively (**Fig 1B**) [8]. Proliferation of GABAergic progenitors depends on the transventricular delivery of SHH produced by the choroid plexus [33]. Purkinje cell progenitors, interneuron progenitors, and astroglial cells are generated from the pancreas specific transcription factor, 1a (*Ptf1a*)-expressing ventricular zone in a temporally overlapping manner [9]. Deletion of *Ptf1a* leads to a global loss of GABAergic subtypes [34] and a fate switch to granule cell progenitors, implying that *Ptf1a* represses the genetic determinants of granule cell identity [35].

**Fig 2:**
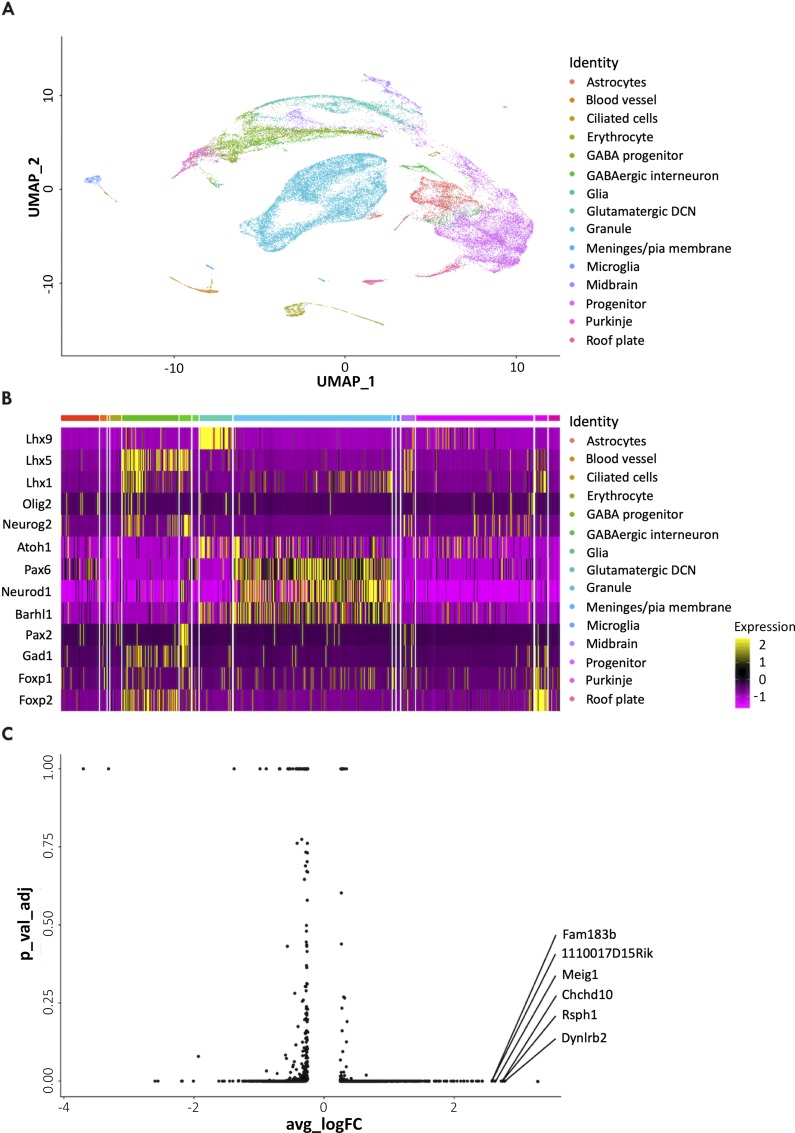
Single-cell characterisation of cellular subtypes in the mouse cerebellum. Data from Carter and colleagues [36] was downloaded from the European Nucleotide Archive (PRJEB23051). In total, 39,245 cells meeting the authors’ quality control cutoffs were projected in UMAP space using Seurat v 3.1.0 according to the embedded metadata. The FindMarkers command was used to perform differential expression testing (ranked Wilcoxon sum test) to distinguish cell type–specific markers. Scaled data were visualised using the DoHeatmap command. (A) UMAP projection of the single-cell data set identifies all known major subtypes of the developing cerebellum. Nonneural cell types are also visualised. Differential gene expression at cellular resolution readily distinguishes the major cellular subtypes of the cerebellum (B) and identifies a novel cell type (C). (B) Heatmap of selected, established transcriptional markers of the respective cell types. Each vertical bar represents a single cell. Column (cell identity) width is proportional to the number of cells present in that cluster. (C) Volcano plot of differential gene expression in a rare cell type labelled ‘ciliated cells’. The labels indicate genes that encode ciliary proteins, which are significantly enriched in this cell type. Atoh1, atonal bHLH transcription factor 1; Barhl1, BarH like homeobox 1; Chchd10, coiled-coil-helix-coiled-coil-helix domain containing 10; DCN, deep cerebellar nuclear neuron; Dynlrb2, dynein light chain roadblock-type 2; Fam183b, family with sequence similarity 183, member B; Foxp, forkhead box P; Gad1, glutamate decarboxylase 1; Lhx, LIM homeobox protein; Meig1, meiosis expressed gene 1; Neurod1, neurogenic differentiation 1; Neurog2, neurogenin 2; Olig2, oligodendrocyte transcription factor 2; Pax, paired box; Rsph1, radial spoke head 1 homolog; UMAP, Uniform Manifold Approximation and Projection.

Within the GABAergic class, Purkinje and interneuron progenitors arise from spatially demarcated dorsoventral regions of the ventricular zone. These major postmitotic cell types are readily identified by the interrogation of a recently published mouse scRNA-seq data set (**Fig 2A**) [36]. In simple terms, algorithms such as Louvain clustering [37] treat cells as ‘nodes’ in a network and measure their relatedness. Clustering algorithms used in conjunction with dimensionality reduction tools (such as t-distributed stochastic neighbour embedding [tSNE] and uniform manifold approximation and projection [UMAP]) can model relationships between distinct cell types. The delineation of all major cell types in this way illustrates the remarkable power of this approach to robustly identify unique cell states without prior knowledge of cellular properties. In line with temporal identity transitions at other levels of the neuraxis [38], cross-repressive interactions between lineage-defining transcription factor GS homeobox 1 (*Gsx1*) and oligodendrocyte transcription factors 1/2 (*Olig1/2*), expressed by interneuron and Purkinje cell progenitors, respectively, are involved in the temporal switch in neuronal identity [39].

The ventricular zone in the mouse can also be partitioned longitudinally into multiple GABAergic domains by combinatorial patterns of transcription factors [16, 40], which is reflected by single-cell transcriptomes [23]. Assigning the cellular origins of distinct postmitotic cerebellar subtypes to the latter progenitor pools is facilitated by single-cell genomics. The presence of cells at varying developmental stages in a single experiment allows statistical techniques such as pseudotemporal ordering to be used to examine lineage commitment [41]. Moreover, pseudotime trajectories permit identification of divergent cell identities from common ancestral progenitors. In this way, mouse scRNA-seq has revealed common progenitors for GABergic and glial cell types [23].

Single-cell clustering algorithms identify cells that constitute the murine cerebellum from embryonic day (E) 10 to postnatal day (P) 10, including astrocytes, blood vessels, microglia, roof plate, GABAergic progenitors, glutamatergic deep cerebellar nuclear cells, and granule and Purkinje cells (**Fig 2A**) [36]. An early group of uncommitted progenitors are identified at E10–12 (‘progenitor’ subtype in **Fig 2A and 2B**) [36]. The latter population is distinguished from committed GABAergic progenitors that emerge from E14 by their differential expression of key cell fate determinants. As they undergo progressive commitment, cells in the GABAergic lineage express known fate determinants in the temporal order predicted by conventional gene expression analyses: initial expression of *Ptf1a*/kirre like nephrin family adhesion molecule 2 (*Kirrel2*) is followed by *Olig2*, LIM homeobox proteins 1/5 (*Lhx1*/*5*), and neurogenin 1/2 (*Neurog1/2*) [23]. The differential expression of these genes in GABA progenitors is captured in a heatmap representation of single-cell gene expression (**Fig 2B**). At this fine-grained level of resolution, a hitherto cryptic domain in the posteriormost ventricular zone was revealed that contained bipotent roof plate–rhombic lip progenitors [23]. How this region is specified and whether inductive signals from adjacent cells or tissues pattern this territory of the ventricular zone is unclear.

The organisation of Purkinje cells and interneurons, the topographic organisation of afferent and efferent projections, and gene expression patterns confer upon the cerebellum a highly compartmental architecture [8, 42, 43]. In the mouse, chronologically labelled ventricular zone Purkinje cell progenitors aggregate into approximately 50 clusters that occupy nested, mediolaterally organised domains that become spatially rearranged into stripes postnatally [43–46]. Refined statistical clustering of a subset of the sampled cell population corresponding to Purkinje cells suggests that there are five Purkinje cell subtypes on the basis of their selective expression of a corresponding number of transcription factors [23]. Numerous previously identified markers that define Purkinje cell clusters are differentially expressed by the latter five subgroups. Clusters also differ in terms of transcription factor dosage, specifically forkhead box P1 (*Foxp1*) and *Foxp2* [23], which are relatively enriched in this cell population overall (**Fig 2B**). To corroborate this finding, Purkinje cells will need to be isolated across a series of embryonic and postnatal time points to identify gene signatures that distinguish subtypes. Likewise, the level of expression of another transcription factor, OLIG2, in the cerebellar oligodendrocyte lineage discriminates between mature oligodendrocytes and immature oligodendrocyte progenitors [47].

Interneuron progenitors contribute GABAergic neurons to the deep cerebellar nuclei and, ultimately, to several distinct interneuron subtypes as they transit through the prospective white matter of the developing cerebellum [9, 48]. Their transcriptional profile overlaps that of Purkinje cells and includes shared expression of *Foxp1/2* (Wizeman and colleagues [2019] [23]), which is corroborated by an independent data set produced by Carter and colleagues (**Fig 2B**) [36]. A key difference is the up-regulation of paired box 2 (*Pax2*) in prospective interneurons (**Fig 2B**) [36]. Further refinement of their identity is dependent on instructive signals from the environment of the prospective white matter [48]. Single-cell analyses at stages during their migration through the prospective white matter should prove informative in understanding how interneuron heterogeneity is sculpted by the environment.

Genetic fate mapping in the mouse has revealed that the upper rhombic lip sequentially generates neurons of the deep cerebellar nuclei, granule progenitor cells, which give rise to the most numerous cell type in the brain, and unipolar brush cells (**Fig 1A and 1B**) [49, 50]. In this species, the rhombic lip can be partitioned molecularly by the differential expression of LIM homeobox transcription factor 1 alpha (LMX1A), wntless Wnt ligand secretion mediator (WNTLESS), atonal bHLH transcription factor 1 (ATOH1), eomesodermin (EOMES), and PAX6 [51, 52]. Progressively later-born granule neurons settle in more-posterior lobes of the cerebellum [49]. This migratory behaviour is linked to an anterior–posterior (AP) orientation of progenitor cell division that greatly expands the cerebellum along that axis [53]. Hypotheses about the regulation of oriented cell division of granule cell precursors include AP striped molecular cues—for example, engrailed 1 (*En1*), *En2*, *Pax2*, *Wnt7b*, and Eph receptor A4 (*EphA4*) [54, 55]—adhesion factor–mediated mechanical constraints [56], and mitotic spindle orientation by the centrosome-associated protein, growth associated protein 43 (GAP-43) [57].

Mature granule neurons are produced in three stages: migration from the rhombic lip is followed by aggregation in a secondary germinal zone, the external granule layer of the cerebellum, where granule cell progenitors become exposed to mitogenic signals, principally SHH from Purkinje cells [58], and massively expand in number; as they exit the cell cycle, granule cell progenitors down-regulate *Atoh1* and migrate deeper into the cerebellum, forming the internal granule layer beneath the Purkinje cell layer [59]. SHH activity and ATOH1 expression are codependent in mouse granule cell progenitors: SHH stabilises ATOH1 through phosphorylation [60]; reciprocally, ATOH1 regulates their SHH responsiveness through ciliogenesis by activating its direct target, centrosomal protein 131 (*Cep131*), which stabilises primary cilia [61].

scRNA-seq provides strong evidence for a common progenitor for granule neuron precursors and glutamatergic deep cerebellar nuclear cells in the mouse [23, 36]. Moreover, the progression of these bipotent cells along the pathway to their alternate fates occurs asynchronously, suggesting a stochastic component to the progressive restriction in progenitor fates. This seems surprising for a lineage branching system that reproducibly generates dichotomous glutamatergic subtypes. One possibility is that these transcriptional differences are not biologically significant, because protein expression in the common progenitors is buffered against fluctuations in mRNA expression. Alternatively, cell fate specification in this lineage could involve a stochastic process to select between binary cell identities [62]. Refined statistical clustering of the subset of glutamatergic cells suggests additional subtype heterogeneity on a scale similar to what is observed in ventricular zone derivative cells [23]. As with Purkinje cell subtypes, there is a relationship between these cell groups and their location in the embryonic cerebellum.

Whether the ‘cell clusters’ identified in these early single-cell analyses persist in the adult is of interest. Recent scRNA-seq studies report 19–48 major cell clusters, with further subtype heterogeneity revealed upon refined clustering [23, 36, 63]. The number of major clusters identified positively correlates with the number of developmental time points sampled in these studies. In all studies, clusters can be grouped by their maturity and lineage; for example, one study with 34 clusters grouped these into five broad cell types [63]. The mapping of clusters to known major cell types remained consistent across studies, demonstrating the robustness of this approach in identifying all major cell types of the cerebellum.

Differential gene expression testing within whole populations on subset clusters of interest is one way to discover and validate biomarkers to distinguish cell types and identify novel, rare cell types. It also holds great promise to explore cell/tissue-specific gene expression indicative of particular pathways. As a basic proof of principle, we subsetted barcoded cells identified by Carter and colleagues [36] as ciliated cells and performed differential expression testing on this population (**Fig 2C**). Enriched genes include dynein light chain roadblock-type 2 (*Dynlrb2*), a target of forkhead box J1 (FOXJ1) that regulates motile ciliogenesis [64], and radial spoke head 1 homolog (*Rsph1*) mutations are a cause of primary ciliary dyskinesia [65]. More fundamentally, whether identified cell clusters represent stable or transient cell states is a key consideration. Reassuringly, a comparison of two independent mouse single-cell transcriptomic data sets showed that cell types identified at early time points persist in the postnatal cerebellum [23]. Moreover, independent corroboration of cell types identified through scRNA-seq by the Allen Developing Mouse Brain Atlas [66] reinforces the validity of scRNA-seq in constructing a cellular taxonomy of the cerebellum [23].

### Plasticity of cerebellar neuronal subtypes

Reflecting their responsiveness to programming by environmental cues, heterochronically transplanted interneuron progenitors acquire fates temporally consistent with the host tissue through whose prospective white matter they migrate [48]. Furthermore, following experimental injury of the external granule layer, Purkinje cell layer stem cells were able to repopulate the lesioned region, proliferate, and switch fates to granule progenitors under the influence of Purkinje cell–derived SHH in mice [67]. How these cells are able to switch their physiological developmental programme and respond differently to the same signal, SHH, is not understood. An attractive hypothesis is that following granule cell injury, the directionality of Purkinje cell–derived SHH signalling is altered, resulting in a concentration-dependent reprogramming of stem cell fate analogous to the specification of ventral spinal cord neuronal identities by graded SHH signalling [68, 69]. Whether the identity of cells in the forming cerebellum could depend on graded SHH signalling emanating from Purkinje cells has not been tested, however. Moreover, the mechanism of cilia formation, which is required for SHH responsiveness in the latter nongranule cells, has not been clarified. The hitherto unappreciated heterogeneity of cerebellar subtypes is further exemplified by a transient population of immature Purkinje cells that only exit the cell cycle in the early postnatal period. These cells also have a cryptic postnatal proliferative capacity, but are lineage restricted and capable of replenishing only Purkinje cell numbers upon injury [70].

### Divergent rodent and human cerebellar neuronal subtype specification

The cerebellum of human and mouse differs in terms of cerebellar morphogenesis, neurogenesis, neuronal subtype ratios, and developmental time line. One of the most striking features in humans in common with a nonhuman primate, the macaque, is the presence of a proliferative subventricular zone (SVZ) in both embryonic cerebellar germinal regions [31]. Uniquely, in humans, rhombic lip substructure persists throughout gestation and remains proliferative postnatally, contributing granule cell progenitors to the posterior vermis. Bulk transcriptional profiling of human rhombic lip identified expression of the basal progenitor marker EOMES in the SVZ, whereas the ventricular zone compartment of the rhombic lip was enriched for Hippo signalling pathway and WNT pathway genes involved in cell growth.

In parallel with the ultrastructural differences between mouse and human germinal zones, genomic methods have been employed to compare cerebellar neurons and glia between these species [47, 71]. Independent analyses using single-nucleus droplet-based sequencing (snDrop-seq) [71] or sequencing of pooled nuclei from distinct subpopulations of cells (nucRNA-seq) [47] showed variable outcomes in identifying major homologous differentiated cell types in human postmortem brain tissue. In particular, the rare Purkinje cell population could not be identified in the former study [71] but was detected using the latter approach [47]. Homologous cell types were found to have common and species-related gene expression profiles [47, 71]. Whereas the core gene regulatory programmes that distinguish specific cell types are conserved across species, global gene expression across these cell types is largely species specific. Overall, homologous neuronal and glial cell types in mouse and human diverge by hundreds of genes that are unrelated to age, gender, or postmortem delay of the human samples. These species-related gene expression differences were confirmed in human and mouse granule and basket interneuron [9] nuclei by assay for transposase-accessible chromatin using sequencing (ATAC-seq) [72]. ATAC signals are strongly associated with the promoters and gene bodies of expressed genes. In general, in both cell types, species-related gene expression matches the genome location of ATAC peaks, whereas repressed genes lack corresponding ATAC peaks [47].

The unique expression profiles in human cerebellar cell types consist of genes that are functionally unrelated [47]. Even for functionally conserved pathways, there is species divergence in the expression of genes within the same family. For example, mouse and human granule cells express two different members of the calcium and calmodulin-dependent phosphodiesterase gene family, which is important for the regulation of neuronal activity. These expression profile differences underscore a divergence of gene regulatory mechanisms that has been proposed to confer new functional properties upon homologous cell types over the course of evolution [47, 73]. Furthermore, genes that are the most divergent in expression between species have the least-conserved regulatory (noncoding) sequences compared with genes that have concordant expression. This implies that evolutionary differences in *cis*-regulatory motifs account for most of the gene expression differences, although differential transcription factor expression can also contribute in some instances [47].

## Conclusion and future perspectives

The apparent simplicity of cerebellar architecture is underpinned by a handful of key transcription factors and signalling molecules that initiate complex gene regulatory networks in the emerging cerebellum. An unexplained, fundamental aspect of the dynamic developmental processes that gives rise to the cerebellum is the switch in temporal identities of progenitor cells. Cross-repressive interactions between key pairs of transcription factors provide an access point to focus investigation of the pathways involved in this process. In general, however, it is difficult to infer causal relationships from single-cell gene regulatory networks that could explain this fascinating property of cerebellar progenitors [41]. Furthermore, the dynamic nature of development makes it hard to determine whether cell clusters identified in scRNA-seq studies represent transient cell states or rare cell populations with stable identities. Single-cell genetic perturbation screens combined with scRNA-seq will be important to determine causality in gene regulatory networks and test the stability of cellular identities under controlled conditions [74, 75]. A basic assumption of current inferred cellular developmental trajectories (pseudotime) is that there is a continuum of cellular states [41]. Combining pseudotime analysis with additional information derived from lineage tracing and spatial information, for example, can uncover converging or diverging pathways in lineage hierarchies, which might not be evident from pseudotime trajectories [76].

The technological advance represented by human and mouse cerebellar organoid differentiation from pluripotent stem cells [77, 78] presents an opportunity to clarify how closely aligned in vivo and in vitro cellular taxonomies are using single-cell genomics [23, 36, 63]. A similar approach in human cerebral organoids revealed cellular differentiation trajectories that are closely matched to human brain, validating their use as platforms to investigate species-related development, evolutionary divergence, and disease [79]. Human postmortem studies [31] should prompt a search for analogous compartmental organisation of cerebellar organoids, specifically whether these models recapitulate an SVZ. The mechanisms underlying cellular plasticity in the early cerebellum [67] are also amenable to investigation using these technologies, which could have translational relevance for neural repair. Integration of human cerebellar organoid scRNA-seq and postmortem nucRNA-seq with complementary epigenetic markers of cell identity should provide a firmer basis for the inference of lineage hierarchies.
